# Cardiotoxicity associated with CTLA4 and PD1 blocking immunotherapy

**DOI:** 10.1186/s40425-016-0152-y

**Published:** 2016-08-16

**Authors:** Lucie Heinzerling, Patrick A. Ott, F. Stephen Hodi, Aliya N. Husain, Azadeh Tajmir-Riahi, Hussein Tawbi, Matthias Pauschinger, Thomas F. Gajewski, Evan J. Lipson, Jason J. Luke

**Affiliations:** 1 University Hospital Erlangen, Erlangen, Germany; 2 Dana Farber Cancer Institute, Boston, MA USA; 3 University of Chicago Comprehensive Cancer Center and Pathology, Chicago, IL USA; 4 University of Texas MD Anderson Center, Houston, TX USA; 5 Paracelsus University Hospital Nuremberg, Nürnberg, Germany; 6 Sidney Kimmel Comprehensive Cancer Center, Baltimore, MD USA; 7 Department of Dermatology, University Hospital Erlangen, Ulmenweg 18, 91054 Erlangen, Germany

**Keywords:** Checkpoint inhibitor, Immune-related adverse events, Melanoma, Immunotherapy, Myocarditis, Cardiomyopathy, Ipilimumab, Nivolumab, Pembrolizumab

## Abstract

Immune-checkpoint blocking antibodies have demonstrated objective antitumor responses in multiple tumor types including melanoma, non-small cell lung cancer (NSCLC), and renal cell cancer (RCC). In melanoma, an increase in overall survival has been demonstrated with anti-CTLA-4 and PD-1 inhibition. However, a plethora of immune-mediated adverse events has been reported with these agents. Immune-mediated cardiotoxicity induced by checkpoint inhibitors has been reported in single cases with variable presentation, including myocarditis and pericarditis.

Among six clinical cancer centers with substantial experience in the administration of immune-checkpoint blocking antibodies, eight cases of immune-related cardiotoxicity after ipilimumab and/or nivolumab/pembrolizumab were identified. Diagnostic findings, treatment and follow-up are reported. A large variety of cardiotoxic events with manifestations such as heart failure, cardiomyopathy, heart block, myocardial fibrosis and myocarditis was documented.

This is the largest case series to date describing cardiotoxicity of immune-checkpoint blocking antibodies. Awareness, monitoring of patients with pre-existing cardiac disorders and prompt evaluation by the treatment team is essential. Treatment including application of steroids is critical for patient safety.

## Introduction

Immune-checkpoint blocking antibodies including anti-CTLA-4 and anti-PD1 can induce tumor responses in various tumor types including melanoma, non-small cell lung cancer (NSCLC), renal cell cancer (RCC), and Hodgkin disease. The anti-CTLA-4 monoclonal antibody ipilimumab and the anti-PD-1 monoclonal antibodies nivolumab and pembrolizumab have been approved by regulatory agencies in several countries for the treatment of metastatic melanoma and are associated with response rates ranging from 10–15 % [1, 2], 31–44 % [3, 4] and 33–38 % [5, 6], respectively. These data have been generated predominantly in cutaneous melanoma however responses have also been observed in rarer melanoma subtypes like uveal [7, 8] or mucosal melanoma [9, 10]. Many of these tumor responses are durable over years [3, 5, 11, 12]. Furthermore, since anti-PD1 antibodies have shown efficacy in other tumor types, leading to approval in NSCLC and RCC, they will increasingly be applied.

Despite immune-checkpoint modulating antibodies revolutionizing clinical immunotherapy, their application is also associated with a spectrum of immune-related adverse events (irAEs) including colitis, endocrinopathies, hepatitis and pneumonitis. The frequency of irAEs varies with any grade toxicity by Common Terminology Criteria for Adverse Events (CTCAE) of 64–80 % of patients (23 % Grade 3/4) treated with ipilimumab [1, 13], up to 79 % (13 % Grade 3/4) in patients treated with pembrolizumab [5] and up to 96 % (55 % Grade 3/4) in patients treated with the combination of ipilimumab and nivolumab [4].

Most organ systems have been observed to be potential targets of immune-related toxicity induced by immune-checkpoint blocking antibodies [5, 14]. Frequent irAEs include colitis, hepatitis, rash and endocrinopathies. Other, less frequent events have also been reported for anti-CTLA-4 antibodies [15–20] as well as anti-PD1 antibodies [10, 21–23]. Particularly, within a retrospective study among 752 patients, one case of ipilimumab-induced myocardial fibrosis [24] has previously been reported. Additionally, selected cases have been reported in the literature including pericarditis [25] and cardiomyopathy with takotsubo-like syndrome [26] induced by ipilimumab and acute heart failure [27] under therapy with pembrolizumab.

To gain a more robust understanding of immune-related cardiotoxicity associated with immune-checkpoint blocking antibodies, we reviewed the experience of several institutions in the United States and Germany with high patient volume treated with these agents. Suspected cases of cardiotoxicity were vetted for alternative explanations and a case series is presented here.

## Methods

Treating physicians in the melanoma community were queried for submission of case reports and institutional databases (with appropriate local Institutional Review Board approval) were reviewed to identify patients who likely experienced immune-related cardiologic adverse events from immune-checkpoint antibody treatment. We identified and assessed in detail a total of twelve patients with cardiac side effects in association with checkpoint inhibitor treatment. In a total of eight patients adverse events were judged to be likely treatment-associated after thorough evaluation of findings (Table 1).

**Tab. 1:** Patients’ characteristics, overview of cardiologic side effects and outcome

Pat-ID	Age (y)	Gender (m/f)	Type of pathology (cardiomyopathy, heart block, etc.)	Occured in week xxx after initiation of checkpoint inhibitor therapy	Signs and symptoms	Treatment of side effect	Outcome of side effect (resolved/permanent changes/other)	Other immune-related AEs	Ipilimumab (number of doses and dosage)	Nivolumab or Pembrolizumab (number of doses and dosage)	Clinical response (CR, PR, MR, SD, PD)	Survival in months from time of distant metastases
1	72	M	myocarditis and cardiomyopathy	week 22	Clinical findings: edema, ascites, pleural effusion, dyspnea	diuresis, steroids (1 mg/kg), life vest	good regression of symptoms under steroid therapy; slight permanent decrease in EF	thyroiditis, hypophysitis	Ipilimumab 3 mg/kg x 4	Nivolumab 1 mg/kg x 4 followed by 3 mg/kg x 6	PR	22
					ECHO: EF from 50 % down to 15 % und up again to 40 %; dilatation of heart							
					Stress MRI: DCM, EF 15-23 %, no signs for ischemia							
					Cardiac catheterization: no signs for ischemia							
					Endomyocardial biopsy: interstitial inflammation mainly with lymphocytes and interstitial fibrosis							
2	68	M	cardiomyophathy	week 12	Clinical findings: dyspnea, edema	diuresis	resolved	none	Ipilimumab 3 mg/kg x 4	no	PD	40+
					Echo: decrease of EF to 46 %; mild LV dysfunction; increased pulmonary pressures with moderate tricuspid regurgitation							
					Endomyocardial biopsy: Myocyte hypertrophy, interstitial and perivascular fibrosis, mild focal subendocardial myocyte vacuolization. Focal fibrous endocardial thickening.							
					Transmission electron microscopy: mild perinuclear accumulation of lysosomes, consistent with lipofuscin pigment deposition; increase of cytoplasmic glycogen and the number of mitochondria, without anomalous forms.							
					Cardiac catheterization: no evidence of coronary artery disease, however, measurements of his right heart pressures suggested an RA pressure of 16, RV pressure of 67/10, wedge of 23 and PA pressures of 68/39							
3	61	M	myocardial fibrosis	week 4	Clinical findings: no cardiologic symptoms	steroids (2 mg/kg), intensive care unit	fatal; massive autoimmune side effects could not be overcome	autoimmune hepatitis	Ipilimumab 3 mg/kg x 2	no	died of side effects	3
					Endomyocardial biopsy: myocardial fibrosis identified at autopsy							
4	81	M	heart failure	week 22	Clinical findings: dyspnea	diuresis	permanent decrease in EF	colitis, hypophysitis	Ipilimumab 3 mg/kg x 3	no	PR	22+
					ECHO: moderately-to-severely reduced left ventricular EF at 35 %; mildly dilated left ventricle; global hypokinesis with regional variation; akinetic basal inferior and inferoseptal segments							
5	23	M	myocarditis/CHF	week 31	Clinical findings: cardiogenic shock requiring dopamine and dobutamine gtt	High dose steroids 2 mg/kg methylprednisolone per day converted to 80 mg prednisone/d with taper over 1 month, ACEi and beta blocker	resolved to baseline (NYHA C1)	uveitis	Ipilimumab 3 mg/kg x 4	no	SD	31
					Endomyocardial biopsy: T cell infiltration without eosinophilia							
					ECHO: drop of EF to 20 %							
					Cardiac MRI: left ventricular dilation and moderate LV systolic dysfunction (LVEF 34 %); right ventricular dilation and moderate RV systolic dysfunction (RVEF 33 %); increased T2 signal in the mid-inferolateral wall, suggestive of underlying myocardial edema supportive of myocarditis.							
6	64	M	myocarditis	week 5	Clinical findings: fatigue, seizures, abdominal pain	dopamine and fentanyl	fatal	none	Ipilimumab 10 mg/kg x 2	no	NA	died of side effects
					Cardiac catheterization and electrocardiogram: normal							
					evidence of myocarditis and LV hypertrophy upon autopsy							
7	88	M	cardiac arrest	week 20	Clinical findings: collapse with cardiac arrest during shopping without any prodromi	AED with defibrillation, intensice care unit, catecholamines, steroids (125 mg i.v./d)	resolved	none	no	Pembrolizumab 2 mg/kg x 9	PR	8+
					ECHO: akinesis of the apex							
					Cardiac catheterization and electrocardiogram: coronary artery disease with no culprit stenosis, reduced LV function; similar to taktsubo cardiomyopathy							
8	80	M	myocarditis	week 5	Clinical findings: dyspnea, edema, arrhythmias	steroids (10 mg dexamethasone + 4 mg every 4 h), intensive care unit	fatal	autoimmune hepatitis	Ipilimumab 3 mg/kg x 2	no	PR	died of side effects
					EKG: atrial fibrillation, right bundle-branch block							
					Nuclear stress test: no stress-induced ischemia							
					ECHO: drop of EF to 31 %, hypokinesis							
					Endomyocardial biopsy: Multinucleated giant cells, lymphocytes, eosinophils							

This retrospective study was approved by the institutional review board of the Friedrich-Alexander-University Erlangen-Nürnberg. In addition, all clinical protocols were reviewed and approved by the local institutional review boards of each participating center and were performed according to Good Clinical Practice (GCP) and the Helsinki Declaration. The approving bodies at the centers were Dana-Farber/Harvard Cancer Center Institutional Review Board, the ethical committee St. Gallen, the institutional review board of the Friedrich-Alexander-University Erlangen-Nürnberg, the institutional review board of the University of Chicago, and the University of Texas MD Anderson Cancer Center IRB.

## Case series

### Case 1 – Myocarditis and cardiomyopathy responding well to steroids

A 72-year-old man with past medical history of myocardial infarction, diabetes mellitus type II, arterial hypertension, peripheral arterial disease stage IIb and hyperuricemia was diagnosed with metastatic *BRAF* wild-type melanoma and treated with ipilimumab (3 mg/kg, 4 infusions) and nivolumab on a clinical trial (1 mg/kg, 4 infusions followed by 3 mg/kg every 2 weeks; CA209067; NCT01927419).

**Fig. 1:**
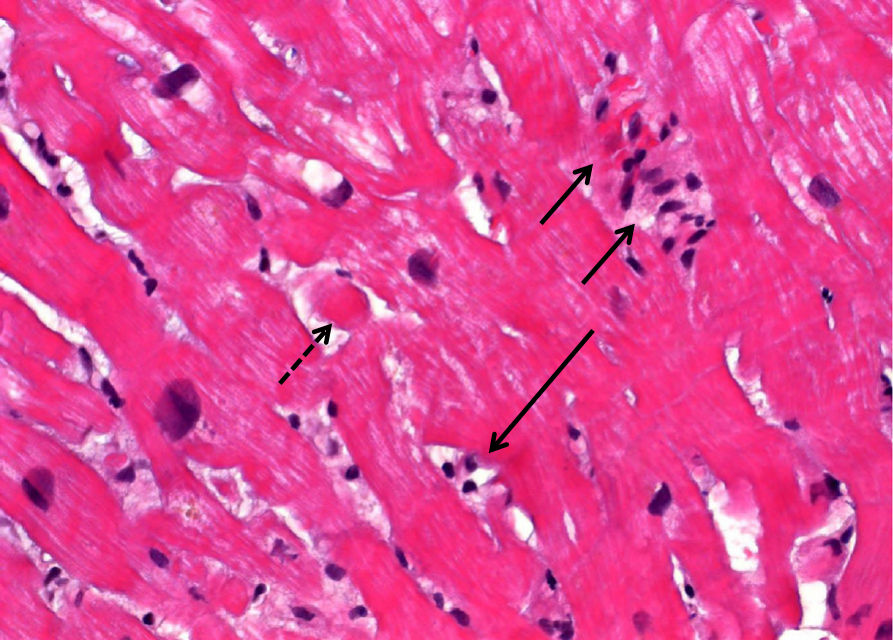
Histologic presentation of case 1. Endomyocardial biopsy shows interstitial fibrosis with some interstitial lymphocytes. Signs of hypertrophy are detectable

After 3 infusions of the combination the patient presented with dyspnea, peripheral edema and anasarca including weight gain of 10 kg. Subsequent diagnostic testing including echocardiography, stress magnetic resonance imaging (MRI) and cardiac catheterization showed a reduction of ejection fraction (EF) from 50 % to 15 % and no suggestion of ischemia. Diuretic medication (furosemide) was started and a life vest fitted. In addition, the patient received an ACE-inhibitor (ramipril 2 × 5 mg/d), metoprolol (2 × 47.5 mg/d) and spironolactone (25 mg/d). Cardiac biopsy revealed interstitial inflammation mainly with lymphocytes and interstitial fibrosis (Fig. 1) with no signs of viral infection. A diagnosis of immune-induced myocarditis was made and corticosteroids were initiated at 1 mg/kg orally. Viral serologies from peripheral blood were investigated with a viral panel being non-reactive for coxsackie virus, adeno- and enteroviruses, EBV and CMV. The patient was previously tested for hepatitis B, C and HIV and found to be non-reactive. Clinical symptoms improved within the first week and ejection fraction increased to 30 % at 10 days of follow up and stabilized two months later at 40 %. Steroids were tapered during this period with frequent clinical follow up and echocardiography. Thus, the initial dose of 80 mg was decreased to 60 mg after two weeks and then reduced by 5 mg every 3–4 weeks depending on improvement of ejection fraction. Other immune-related toxicity experienced by this patient included an autoimmune-thyroiditis with development of thyroid-peroxidase (TPO) antibodies treated with carbimazol, hypothyroidism substituted by 50 μg levothyroxine and hypophysitis managed with physiologic steroid replacement.

The patient had a mixed response to immunotherapy at the first follow-up staging which evolved into a partial response which remained stable over 9 months before the development of progressive disease.

### Case 2 – Cardiomyopathy

A 68-year-old man with a past medical history of clinically asymptomatic dilated cardiomyopathy and alcohol abuse was diagnosed with metastatic *BRAF* wild-type melanoma metastatic to lymph nodes and small bowel in June of 2011 and subsequently treated with 4 doses of ipilimumab between July and September of 2011.

Approximately one month after the last dose of ipilimumab the patient developed dyspnea upon exertion in addition to upper and lower extremity edema, leading to hospitalization for heart failure exacerbation in November of 2011. An initial echocardiogram revealed enlargement of the left ventricle with an EF of 46 %, thickening of the mitral valve, and severe mitral regurgitation, mild to moderate tricuspid regurgitation with severe pulmonary hypertension. Coronary artery disease was ruled out by nuclear cardiac stress test. He was treated with diuretics, beta blocker, and ACE inhibitor with symptomatic improvement and normalization of the volume overload. The etiology was initially attributed to ethanol (ETOH) abuse, however a repeat echocardiogram in December of 2011 performed after strict abstinence from ETOH showed worsened EF (25–30 %), which prompted a right and left heart and coronary catheterization as well as cardiac biopsy. Coronary artery disease was definitively ruled out and measurements of right heart pressures suggested elevated right atrial, right ventricle, and pulmonary artery pressure. A cardiac biopsy was nonspecific but ruled out acute myocarditis. Corticosteroids were not administered and the patient’s performance status only slowly improved over months.

Restaging imaging following completion of ipilimumab treatment demonstrated clear progressive disease. Subsequent oncologic treatment was complicated by multiple hospitalizations due to recurrent cellulitis however the patient was eventually treated with temozolomide chemotherapy. Over the course of 18 months, the patient developed long-term disease stabilization. The patient is alive more than five years following diagnosis of metastatic melanoma. While an association of heart failure exacerbation and treatment with ipilimumab was not definitively established, the close temporal relation, absence of clear other exacerbating etiologies and long-term survival of the patient suggest an immune-mediated etiology triggered by ipilimumab as the most likely culprit of the heart failure.

### Case 3 – Myocardial fibrosis

A 71-year-old man with no significant past medical history other than metastatic melanoma developed steroid-refractory hepatitis shortly after the second infusion of ipilimumab. It was not possible to determine other causes of fibrosis. Ejection fraction had been 65 % before start of therapy. He subsequently developed multi-organ failure with capillary leak, renal insufficiency, and neurological symptoms without brain metastases or haemorrhage. The patient died of multi-organ failure despite immediate induction of high dose steroids and supportive measures in the intensive care unit. Though there were no obvious cardiac symptoms beyond multi-organ failure, autopsy revealed myocardial fibrosis surrounded by structural changes of cardiomyocytes.

### Case 4 – Heart failure

An 81-year-old man with past medical history including atrial fibrillation, coronary artery disease s/p myocardial infarction and ventricular tachycardia/ventricular fibrillation s/p automatic implantable cardioverter-defibrillator placement was diagnosed with metastatic *BRAF* wild-type melanoma. Ipilimumab was initiated however two weeks after the third dose, the patient developed diarrhea deemed immune-related (confirmed by colonoscopy and muscosal biopsy). An 8 week course of steroids and two doses of infliximab eventually lead to resolution of the colitis. In addition, the patient experienced immune-related hypophysitis with secondary adrenal insufficiency and hypothyroidism. Management of colitis was complicated by bacterial pneumonia requiring two hospitalizations but eventually resolved.

Eleven weeks following the third and last dose of ipilimumab the patient developed progressive subacute shortness of breath. An extensive work-up including a computed tomography (CT) of the chest, bronchoscopy, transbronchial biopsy, and bacterial/fungal cultures ruled out a recurrent infectious etiology, other respiratory etiologies, and metastatic disease. Echocardiography showed a left ventricular EF moderately-to-severely reduced at 35 % from a baseline of 47 % as measured by myocardial perfusion single-photon emission computed tomography 14 months prior. The left ventricle was mildly dilated with normal left ventricular wall thickness. On physical examination the patient had an irregularly irregular heart rhythm, mild pitting edema, and bilateral basilar crackles in the lungs. He did not exhibit anginal symptoms. Cardiac enzymes and troponins were negative. He was treated with diuretics resulting in complete resolution of his respiratory symptoms. Though the patient clinically improved over time with diuretics, cardiac function did not return to normal with subsequent echocardiography documenting persistently reduced ejection fraction. Treatment with corticosteroids was not given due to the patient’s clinical improvement with diuretics and previous toxicity from a prolonged steroid course for his immune-related colitis.

Restaging revealed initial tumor shrinkage however subsequent slow progression over an extended period of time. The patient went without treatment for over a year before initiating anti-PD1 antibody treatment with pembrolizumab. No further cardiac events were observed while receiving pembrolizumab.

### Case 5 – Myocarditis/heart failure

A 23-year-old man with *BRAF*^*V600E*^ mutant melanoma received four doses of ipilimumab. The patient had an initial response to treatment however by approximately month 5 after starting ipilimumab, he developed disease progression. Thereafter, the patient initiated vemurafenib. The dose of vemurafenib was reduced from 960 mg daily to 720 mg daily secondary to multiple episodes of uveitis (treated with relief by intraocular steroids).

At approximately month 7 after initiating ipilimumab and 2 months after initiating vemurafenib, the patient developed severe chest pain and cough. Diagnosis of cardiogenic shock was made based on vital sign changes as well as electrocardiogram showing ST segment elevation and elevated cardiac enzymes including creatine kinase and troponin-T. The patient was started on both cardiac inotropic and vasopressor agents and anti-coagulated in the setting of intermittent atrial fibrillation. Transthoracic echocardiogram was performed revealing normal left ventricular size but severe global hypokinesis, left ventricular EF of 20 %, and right ventricular systolic pressure of 33 mm Hg. Diuresis was attempted with intravenous furosemide and cardiac rate and pressure control were obtained. Cardiac MRI was performed and deemed suggestive of myocarditis.

**Fig. 2:**
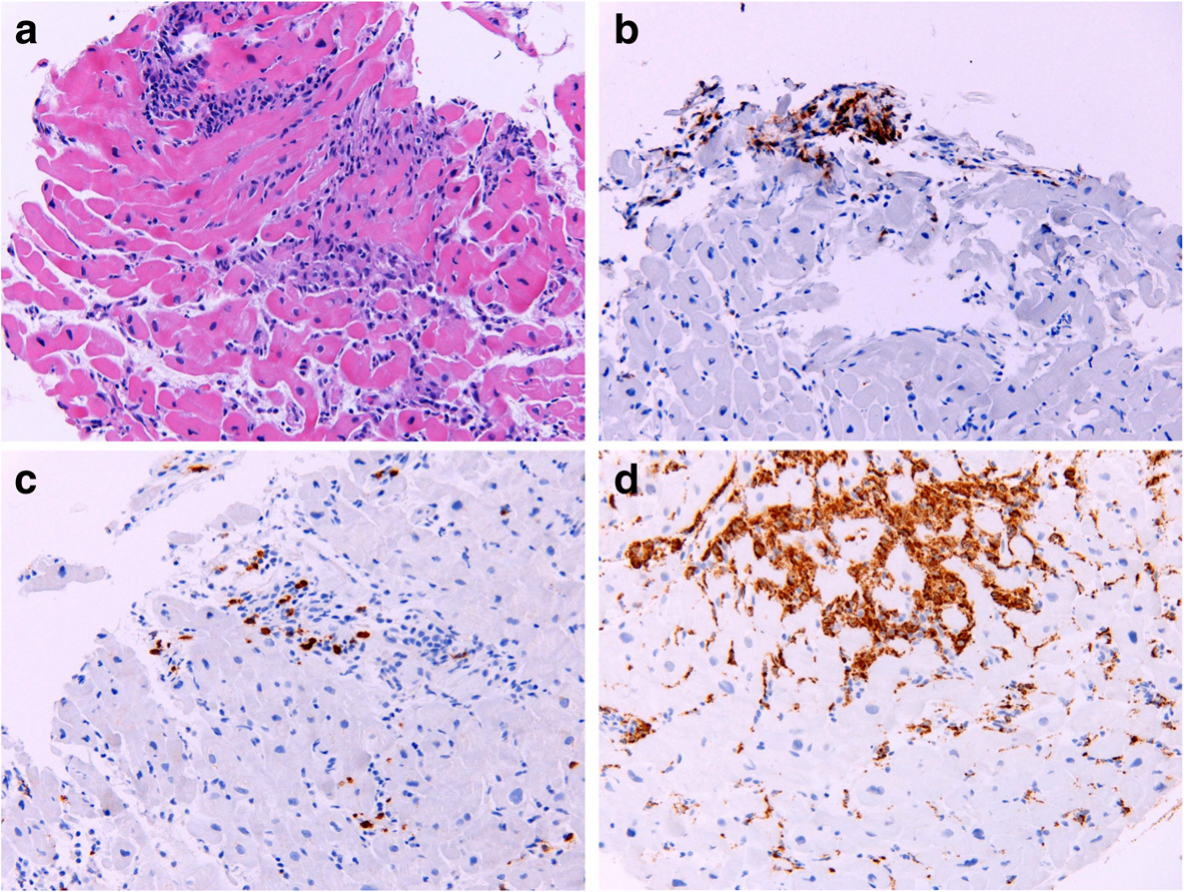
Histologic presentation of case 5. **a** H&E stain of the endomyocardial biopsy shows patchy lymphohistiocytic infiltrates associated with myocyte damage, diagnostic for lymphocytic myocarditis. **b** IHC stain for CD3 highlights in brown T cells within the inflammatory infiltrate (**c**) IHC stain for CD8 highlights in brown T cells within the inflammatory infiltrate (**d**) IHC stain for CD68 shows many histiocytes/macrophages within the myocardial inflammation

After stabilization, right heart catheterization to assess hemodynamics and right ventricular endomyocardial biopsy were performed. Pathology revealed lymphocytic myocarditis manifesting with lymphohistiocytic infiltrates and cardiomyocyte damage without identification of eosinophils (Fig. 2). Electron microscopy confirmed ultrastructural findings consistent with lymphohistiocytic myocarditis. A diagnosis of immune-mediated myocarditis was made and high dose intravenous corticosteroids were initiated until vasopressive agents could be withdrawn. Oral steroids were then tapered over approximately six weeks.

Vemurafenib had been held since admission however this was restarted five days after admission to the hospital. Repeat echocardiograms were performed throughout the steroid taper and the patient was noted to have recovery of EF to baseline with resolution of cardiac wall akinesis within one month of the event. The patient continued on vemurafenib without other significant toxicity until the development of diffuse and multifocal brain metastases approximately one year later. At that time he was treated with whole brain radiation followed by vemurafenib with temozolomide. Despite additional stereotactic radiosurgery to multiple lesions, disease control in the brain could not be obtained and the patient eventually passed away due to progressive melanoma.

### Case 6 – Myocarditis

A 64-year-old man with metastatic melanoma and a past medical history including peripheral vascular disease developed cardiopulmonary failure two weeks following the second dose of ipilimumab. He had no prior cardiac history.

Work-up during admission revealed a slightly diminished creatinine clearance of 86 ml/min (reference range > 90 ml/min), an elevated creatinine of 135 μmol/l (reference range 59–104 μmol/l) and elevated coagulation factor values. However, troponin was normal and coronary angiography unremarkable. Resuscitation was initially performed using dopamine. No corticosteroids were given and the patient passed away five days following initiation of these symptoms. Autopsy revealed cardiac changes consistent with a myocarditis including evidence of left ventricular hypertrophy with fibrosis and microscopic findings including several scattered foci of lymphocytes and eosinophilic granulocytes.

### Case 7 – Cardiac arrest

An 88-year-old man with coronary artery disease was diagnosed with metastatic melanoma. Pembrolizumab was initiated and induced a partial response. The patient developed myalgia and pain in the shoulder after the 8th infusion that was responsive to 5 mg of prednisolone. Four days after the 9th infusion he collapsed while shopping and was immediately resuscitated. He had to be defibrillated four times, intubated and was put on a respirator in an intensive care unit. Diagnostics including echocardiogram and coronary angiography showed akinesis of the apex, coronary artery disease with no culprit stenosis and a reduced left ventricular function of 45 % similar to takotsubo cardiomyopathy. Troponin was initially increased to 0.454 ng/ml, peaked at 2.86 ng/ml and was at 0.697 ng/ml two weeks after the incident (normal range <0.014 ng/ml). CK was 303 U/l upon admittance, maxed at 1198 U/l (normal range 0–170 U/l) and normalized by day 14. He was treated with high dose systemic corticosteroids (125 mg i.v. for 4 days) and subsequently recovered. He is currently taking care of his wife who has dementia in their home and has no progression of melanoma. Anti-PD1 therapy was not re-initiated and staging 3 months after the cardiac arrest without any further tumor therapy showed stabilization of disease.

### Case 8 – Fatal myocarditis

An 80-year-old patient with a previous excision of a primary melanoma was diagnosed with low-grade non-Hodgkin’s lymphoma. He was followed without treatment for 2 years before being treated with 4 doses of rituximab for splenomegaly and minimal FDG avidity in the bilateral axillae without lymphadenopathy. One year later, biopsy of a lung nodule confirmed metastatic melanoma with left hilar and perihilar masses, as well as paratracheal and subcarinal lymphadenopathy and lesions in the rectus muscle wild-type for BRAF, NRAS, and KIT. At that time he had no congestive heart failure and hypertension was mild and well-controlled with one anti-hypertensive. The patient received 2 doses of ipilimumab at 3 mg/kg and presented 2 weeks later with congestive heart failure and autoimmune hepatitis with elevated transaminases and a negative viral hepatitis panel. He was started on intravenous methylprednisolone at 1 mg/kg. His left ventricular ejection fraction was 40 % - 45 % and stage III diastolic dysfunction with restrictive pattern. Congestive heart failure and hepatotoxicity were attributed to ipilimumab treatment. He was discharged to complete levofloxacin therapy for possible left lower lobe pneumonia and to continue prednisone 60 mg by mouth daily.

He was admitted 2–3 days later with shortness of breath. Chest CT revealed a partial response with decrease of pulmonary and mediastinal metastases, but a new small left pleural effusion. He had atrial fibrillation, right bundle-branch block with left anterior fascicular block, non-ST elevated myocardial infarction, and questionable myocarditis. Nuclear stress test showed a fixed perfusion defect in the anterior wall on both stress and rest images consistent with prior myocardial infarction with no reversibility to suggest stress-induced ischemia. There was hypokinesis of the anterior wall and ejection fraction was 31 %. He was initiated on carvedilol 3.125 mg twice daily, and to continue clopidogrel, aspirin, and lisinopril. Additionally, he received metoprolol as needed. The significant bilateral lower extremity edema was treated with furosemide. The patient’s cardiac enzymes reached a troponin of 11.96, CK-MB of 59.9, and CK of 97. He appeared more dyspneic. EKG showed atrial fibrillation and a right bundle-branch block with anterior fascicular block and extensive infarction or myocarditis. Therefore, dexamethasone 10 mg intravenous followed by 4 mg every 6 h was administered. The patient did not tolerate a cardiac MRI. He began decompensating quickly and his blood pressure dropped precipitously. He started experiencing ventricular arrhythmias starting with ventricular tachycardia. He was started on vasopressors and upon arrival to the intensive care unit, his rhythm degenerated into ventricular fibrillation and subsequent asystole and the patient expired.

A post-mortem examination revealed diffuse mottling of the myocardium most prominent in the left ventricle. Microscopic examination revealed diffuse myocarditis with multinucleated giant cells, lymphocytes, and eosinophils, most prominent in the left ventricle and interventricular septum.

## Discussion

The case series presented here reports on the induction of cardiotoxicity by ipilimumab and/or anti-PD1 antibodies. Though such toxicity is rare, multiple manifestations of immune-related cardiac syndromes can be observed. Cardiac fibrosis induced by ipilimumab had been previously examined by one of our groups [24] and thus prompted us to screen for cardiac side effects in checkpoint inhibitor therapy in six large cancer centers. We documented cases of autoimmune myocarditis, cardiomyopathy, heart failure, cardiac fibrosis and cardiac arrest. Pre-existent cardiac pathology or peripheral arterial disease was present in the majority of the patients (5 out of 8). Thus, possibly these cases present a worsening of previous cardiac disorders. However, all of the patients were free of symptoms when starting checkpoint inhibitor therapy. Some of the pre-existent conditions had been stable for decades, i.e. a patient with myocardial infarction 20 years prior to initiating treatment. Corticosteroids improved symptoms when applied. Upon administration of corticosteroids, one patient experienced rapid improvement of ejection fraction that was severely reduced to 15 % due to autoimmune myocarditis. In 5 of the 8 patients (63 %) other organ systems were also affected by immune-related side effects including autoimmune thyroiditis, uveitis, colitis, hepatitis and hypophysitis. It has previously been reported that two or more organ systems were affected in 7 % of patients treated with ipilimumab and in 31 % treated with the combination of ipilimumab and nivolumab (Hodi et al. ASCO 2015; Abstract 9004). Thus, special attention needs to be focused on patients who have already experienced one immune-related adverse event. There have been reports with increased response rates and median survival times in patients with irAE [24]. In this case series, mean survival was 21 months with the patients surviving the severe side effects showing long-term survival.

This study reports on cardiotoxic effects in patients that were treated either with anti-CTLA-4 antibodies or anti-PD1 antibodies. PD-1 is known to protect against tissue inflammation and myocyte damage. A preclinical study in two models of myocarditis showed enhanced disease with increased inflammation and cytotoxic activity in mice with PD1^−/−^ CD8^+^ T cells compared to mice with PD1^+/+^ CD8^+^ T cells [28]. PD-1 deficiency has been described to predispose for spontaneous myocarditis in mice [29, 30] as well as spontaneous dilated cardiomyopathy caused by antibodies to cardiac troponin [31, 32]. CTLA-4 deficient mice are known to rapidly develop multi-organ lymphocytic infiltration with severe myocarditis [33] and in another murine myocarditis model CTLA-4^−/−^ cytotoxic T lymphocytes were more pathogenic than CTLA-4^+/+^ in inducing myocarditis [34]. Thus, the cardiotoxic effects induced by checkpoint inhibitors could be explained by lowering the threshold for activation of T cells specific for self-antigens in the heart.

Cardiotoxicity has been described with other immunotherapies: Cellular based therapies such as autologous anti-MAGE-A3 T lymphocytes have been associated with fatalities due to immune reactions towards myocytes. In these instances, the hypothesized mechanism included cross-reactivity of high affinity-enhanced T lymphocytes with unrelated antigens leading to cardiogenic shock and subsequent death within a few days of infusion of the engineered T cells [35].

The mechanisms of immune-related adverse events resulting from checkpoint inhibitor therapy have not been fully elucidated. For instance, hypophysitis due to ipilimumab could possibly be explained by the expression of CTLA-4 in normal pituitary gland as shown in murine studies [36]. Immune-related adverse events are typically associated with lymphocyte infiltration of the affected organ, for example colon or liver. However, predictive biomarkers that would help to identify which patients and what organ system is at risk for immune-related toxicity are lacking.

Checkpoint inhibitor therapy can induce a spectrum of cardiac side effects. These are rare events – however, physicians treating patients with checkpoint inhibitors should be aware of their potentially cardiotoxic effects. Patients with preexisting cardiac conditions should be closely monitored for deterioration of heart function. The prompt and adequate management of cardiac side effects including application of steroids is critical to avoid a potentially fatal outcome.

## Abbreviations

CT, computed tomography; EF, ejection fraction; ETOH, ethanol; IHC, immunohistochemical; MRI, magnetic resonance imaging; TPO, thyroid-peroxidase; CHF, congestive heart failure
